# Transport of Folded Proteins by the Tat System

**DOI:** 10.1007/s10930-019-09859-y

**Published:** 2019-08-10

**Authors:** Kelly M. Frain, Colin Robinson, Jan Maarten van Dijl

**Affiliations:** 1 The School of Biosciences, University of Kent, Canterbury, CT2 7NZ UK; 2 Department of Medical Microbiology, University Medical Center Groningen, University of Groningen (UMCG), Hanzeplein 1, P.O. Box 30001, 9700 RB Groningen, The Netherlands

**Keywords:** TatA, TatB, TatC, Twin-arginine, *Bacillus subtilis*, *Escherichia coli*

## Abstract

The twin-arginine protein translocation (Tat) system has been characterized in bacteria, archaea and the chloroplast thylakoidal membrane. This system is distinct from other protein transport systems with respect to two key features. Firstly, it accepts cargo proteins with an N-terminal signal peptide that carries the canonical twin-arginine motif, which is essential for transport. Second, the Tat system only accepts and translocates fully folded cargo proteins across the respective membrane. Here, we review the core essential features of folded protein transport via the bacterial Tat system, using the three-component TatABC system of *Escherichia coli* and the two-component TatAC systems of *Bacillus subtilis* as the main examples. In particular, we address features of twin-arginine signal peptides, the essential Tat components and how they assemble into different complexes, mechanistic features and energetics of Tat-dependent protein translocation, cytoplasmic chaperoning of Tat cargo proteins, and the remarkable proofreading capabilities of the Tat system. In doing so, we present the current state of our understanding of Tat-dependent protein translocation across biological membranes, which may serve as a lead for future investigations.

## Introduction

To function correctly and efficiently, every cell needs to be highly organised, tightly regulated and compartmentalised. Proteins are essential macromolecules synthesised by ribosomes in the cytoplasm that often require localisation to a particular subcellular compartment before they can carry out their respective functions. Their proper formation, targeting and activity are imperative to the survival of the cell. This requirement for correct localisation particularly applies to proteins that take part in the acquisition of nutrients, energy transduction, cell-to-cell communication and cellular locomotion. On average, 20–30% of proteins synthesised in the bacterial cytoplasm are destined for extra-cytoplasmic locations [1]. They therefore have to pass a cell membrane composed of a tightly sealed lipid bilayer intent on keeping the cell structurally sound and impenetrable. Therefore, specialised transport systems have evolved within the cell membrane to allow proteins to cross this barrier. Each system made up of critical components is as specialised as the protein cargo it will transport. However common features tie protein transport systems together, which guarantee cell regulation and safety. These include a gated pore, an energy requirement to drive cargo proteins through the membrane, and the use of signal peptides that direct the cargo protein to the correct translocase and the correct location.

**Fig. 1:**
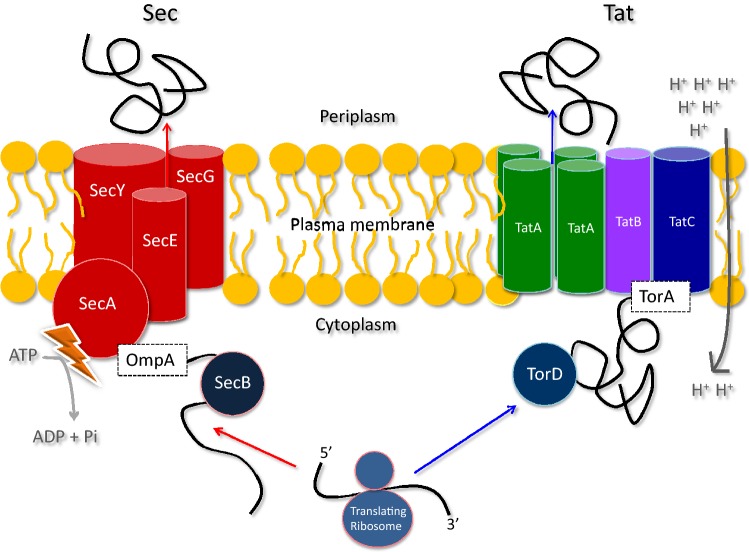
The Sec- and Tat-dependent protein transport pathways. The Sec pathway is the dominant pathway for protein export from the bacterial cytoplasm. It accepts and translocates cargo proteins across the plasma membrane in a loosely folded or unfolded state, here exemplified with the precursor of the outer membrane protein A of *E. coli* (OmpA). Targeting and folding control of the cargo protein is supported by cytoplasmic targeting factors, such as SecB. The Sec machinery itself is composed of the SecYEG channel and the translocation ATPase SecA, which converts chemical energy in the form of ATP into a driving force that pushes the cargo protein through the membrane. Additionally, translocation may be powered by the transmembrane proton gradient. At the *trans*-side of the membrane, the translocated protein folds into its active and protease-resistant final conformation. In contrast to the Sec pathway, the Tat pathway transports fully folded cofactor-containing proteins across the membrane, here exemplified with the precursor of the Tat cargo TorA. Cofactor insertion and folding may be aided by Redox Enzyme Maturation Proteins (REMPS), such as TorD in the case of TorA. The Tat translocase may consist of the three components TatA, TatB and TatC (*E. coli*), or of TatA and TatC components only (*B. subtilis*). Protein transport via Tat is powered by the transmembrane proton-motive force

Two major transport systems exist for protein translocation across the bacterial cytoplasmic membrane, namely the general secretory (Sec) pathway and the twin-arginine translocation (Tat) pathway (Fig. 1). The Sec pathway facilitates export of the majority of bacterial proteins, whereas the Tat pathway is quite restricted. For instance, it transports ~ 30 proteins in *Escherichia coli* and only four in *Bacillus subtilis* [2]. Further, each protein is fully folded in the cytoplasm prior to export via Tat, whereas Sec can only export unfolded proteins.

## Protein Targeting Via the Twin-Arginine Signal Peptide

**Fig. 2:**
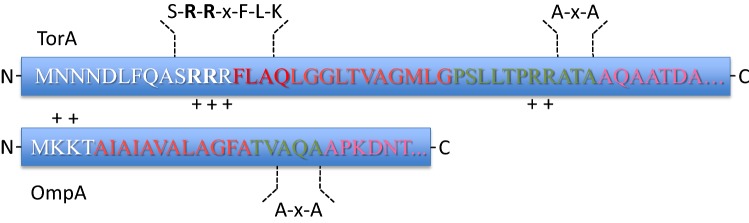
Sec- and Tat-specific signal peptides. N-terminal signal peptides direct proteins to the Sec and Tat translocases in the membrane. They have a conserved tripartite structure, consisting of a positively charged N-region (indicated by ‘white residues’ in one-letter code), a hydrophobic H-region (red) and a C-region (green) that contains the Ala-X-Ala recognition site for signal peptidase. Cleavage by signal peptidase, C-terminally from the Ala-X-Ala sequence, liberates the mature protein (pink) from the membrane. Twin-arginine signal peptides, as exemplified by the TorA signal peptide, contain the canonical twin-arginine motif at the interface of the N- and C-regions. Their H-region is longer and less hydrophobic than that of Sec-type signal peptides, and N-terminally of the C-region there are often positively charged residues that serve in Sec-avoidance. Notably, Sec-type signal peptides, here exemplified by the OmpA signal peptide, are usually much shorter than twin-arginine signal peptides

To ensure proteins are appropriately directed into the Sec or Tat pathways and to initiate the translocation process, specific signal peptides are present on the N-terminus of each protein. On the *trans* side of the membrane the signal peptide is cleaved by a signal peptidase to liberate just the mature protein [3–7]. The amino acid sequences of signal peptides differ substantially, but they are all composed of a positively charged N-terminal N-domain, a hydrophobic H-domain and a C-terminal C-domain with an Ala-x-Ala signal peptidase cleavage site [3, 8] (Fig. 2). Further, the N-regions of Tat signal peptides contain the canonical twin-arginine motif S-R-R-x-F-L-K (where x is a polar amino acid) [9]. The importance of additional conserved amino acids in the Tat-motif depends on the cargo protein and varies in different bacteria [10]. However, RR-residues are close to invariant and key to efficient protein export. In particular, the charge-neutral substitution of RR to KK blocks Tat export completely [11]. Yet, a single Arg to Lys mutation only slows down the rate of translocation in most bacteria [12]. In chloroplast thylakoids where the Tat pathway also exists, an RR to KR substitution is tolerated, while a RR to RK substitution precludes transport [12–14]. A single substitution of Arg to Glu has been reported as tolerated too [15]. Of note, the TtrB subunit of the tetrathionate reductase in *Enterobacteriaceae* is the only known native Tat cargo to have a KR-motif [16]. Aside from the RR-motif, other residues within the larger twin-arginine signal peptide are also important. In particular, the Phe residue is present in 80% of Tat-motifs, and substitutions showed a highly hydrophobic residue is essential at this position [11].

Tat signal peptides comprise about 30 residues in most organisms. Hence they are longer than Sec signal peptides, which comprise about 17 to 24 residues [17]. Tat signal peptides are also overall less hydrophobic than Sec signal peptides, which serves to avoid protein targeting to the Sec pathway [18]. Additionally, the C-domain of Tat signal peptides may include basic residues N-terminally of the A-x-A motif, which contribute to Sec avoidance (Fig. 2) [19, 20].

## The Twin-Arginine Translocation Pathway

In the early 1990’s, an alternative translocase was discovered in the thylakoid membrane of chloroplasts, which worked in parallel to the Sec pathway [21]. Initially this pathway was named the ΔpH-dependent pathway due to its unusual sole requirement of a transmembrane proton gradient for translocation [22]. Three membrane proteins were soon identified in thylakoids as essential for translocation of fully folded proteins via the ΔpH-dependent pathway [23], namely Tha4 [24], Hcf106 [25] and cpTatC [26]. Subsequently, homologous proteins were identified in some bacteria, archaea and even mitochondria [27, 28]. In *E. coli*, the homologues of Tha4, Hcf106 and cpTatC were also shown to be required for export of proteins with twin-arginine signal peptides and, therefore, they were respectively named TatA, TatB and TatC [9, 29–31].

Combined studies on the thylakoidal and bacterial Tat pathways showed that their function is to transport a subset of complex fully folded proteins that require cofactor insertion or immediate oligomerisation [8, 32]. Today, Tat-translocated proteins have been shown to participate in many processes including energy metabolism, cell division, cell envelope biogenesis, quorum sensing, motility, symbiosis and pathogenesis [33–36]. Tat can even export complex heterologous proteins that are Sec-incompatible, like the tightly folded dihydrofolate reductase with bound methotrexate [37], the green fluorescent protein (GFP) [38], and several bio-pharmaceutically relevant human proteins [39]. Another intriguing attribute of the Tat pathway is that it can detect unfolded or mutated proteins, and reject them for export [40, 41].

Based on the number of Tat components involved in protein translocation, essentially two types of ‘translocases’ can be distinguished. The prototype Tat translocase that is active in thylakoids and *E. coli*, consists of the afore-mentioned TatABC components. Further, the minimal Tat translocases, as typified in *Bacillus* species consist of TatA and TatC components only. The types of translocases will be discussed in the following paragraphs.

## The *E. coli* Tat Translocase

The *E. coli tatABCD* operon encodes the core components of this bacterium’s Tat system (Table 1). All four genes are constitutively expressed, but the expression level of *tatA* exceeds that of *tatB* 25-fold that of *tatC* 50-fold [42]. This difference is mirrored in the final component make-up of the Tat translocase in the plasma membrane. The *tatE* gene is constitutively expressed from another chromosomal locus. The *tatB* and *tatE* genes are thought to originate from gene duplications of *tatA* [28, 43]. Although Δ*tatABCDE* strains are viable, the mutants show various defects including impaired septation, decreased motility and an increased sensitivity to detergent [44].

**Table 1:** Comparison of *E. coli* and *B. subtilis* Tat proteins and Tat complexes including their estimated molecular masses (kDa)

*E. coli*				*B. subtilis*			
Protein/complex	Gene product molecular mass (kDa)	Complex molecular mass (kDa)	Ref.	Protein/complex	Gene product molecular mass (kDa)	Complex molecular mass (kDa)	Ref.
TatA	9.6	100–500	[114]	TatAd	7.4	160/270	[77]
				TatAy	6	200	[159]
TatE	7		[43]	TatAc	6.7	100	[78]
TatB	18.4	< 100	[111]				
TatC	28.9	220	[111]	TatCd	28	66–100	[78]
				TatCy	28.9	66	[78]
TatBC		430	[111]	TatAdCd		230/350	[77]
TatABC		580	[104]	TatAyCy		200	[159]
TatABC + substrate		600	[104]	TatAcCd		230	[78]
				TatAcCy		200	[78]

**Fig. 3:**
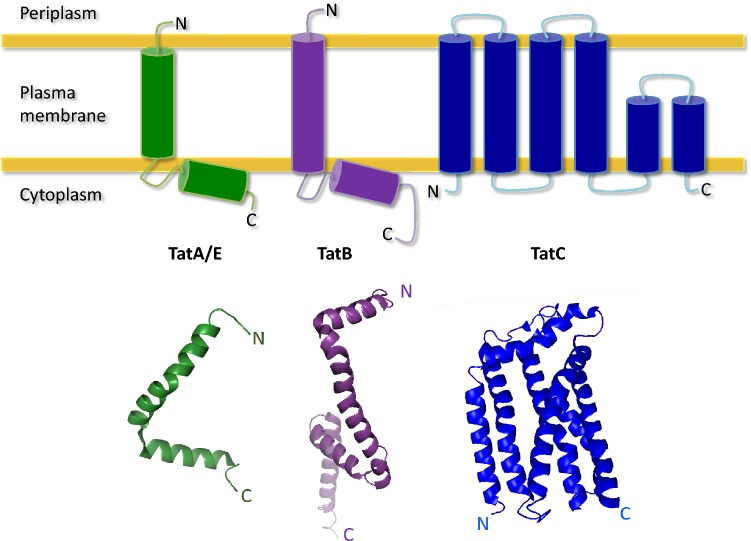
Membrane topology and structures of the TatA, TatB and TatC proteins. The Tat translocase of *E. coli* consists of three components, namely TatA, TatB and TatC. TatB and TatC form a receptor complex for cargo proteins, whereas TatA is the main facilitator for protein translocation across the membrane. TatB is missing from the two-component Tat translocases as encountered in *B. subtilis*. The upper half of the Figure shows a traditional representation of the membrane topology of TatA/E, TatB and TatC based on molecular biological analyses. The lower half of the Figure shows ribbon presentations of the structures of TatA, TatB and TatC as adopted from the RCSB Protein Data Bank (http://www.rcsb.org/structure/). These structures have the following PDB accession codes: TatA—2LZR (solution NMR structure [48]); TatB—2MI2 (solution NMR structure [54]); and TatC—4HTS (crystal structure [63])

TatA (9.6 kDa) is the most abundant component of the Tat complex, most likely responsible for forming the translocase channel [45]. *E. coli* has a core TatA protein, but it also involves the TatA-like proteins, TatB and TatE [28]. Of note, TatE can substitute TatA [43]. TatA, TatB and TatE are similar in structure with a short N-terminal domain that is exposed to the periplasm [46], a single transmembrane helix, an amphipathic helix in the cytoplasm [47], and an unstructured cytoplasmic C-domain [48] (Fig. 3). Surprisingly, not many mutations in TatA block export, but there are a few instances. In particular, Gly33 in the “hinge region” is critical for TatA function [49], and the transmembrane helix and various residues in the amphipathic helix are also important [50, 51].

TatE (7 kDa) is a much smaller than TatA [9]. Given the smaller size and ~ 100-fold lower abundance than TatA, it was initially believed TatE has no real function in the Tat translocon [42]. More recently however, it was shown TatE could substitute TatA [43], and that it is recruited to the Tat translocase [52]. Importantly, TatE was shown to interact with the Tat signal peptide and to even partially prevent premature cleavage of the TorA signal peptide [53].

The role of TatB (18.4 kDa) is to bind the Tat signal peptide and, thereafter, the mature protein. Despite only sharing 20% sequence identity to TatA and being nearly double TatA’s size, TatB is predicted to have a very similar structure and topology (Fig. 3) [50]. Specifically, TatB has a slightly longer amphipathic helix and a longer unstructured C-terminal region [54, 55]. Mutations in TatB’s hinge region and amphipathic helix cause translocation defects [56]. Of note, particular amino acid substitutions in TatA’s N-terminus allow replacement of TatB by TatA [57] [58], supporting the notion that TatB originated from TatA and subsequently specialized [5, 59].

TatC is the largest (28.9 kDa) and best-conserved protein in the Tat complex that aids cargo binding [60, 61]. The structure of TatC is very different to other Tat components as it has 6 transmembrane helices and an N-in C-in topology (Fig. 3) [62]. The crystallisation of TatC from *Aquifex aeolicus*, which shares 40% sequence identity to *E. coli* Tat C, revealed the relative positions of the transmembrane domains [63]. Together, they take the shape of a baseball glove or cupped hand with very restricted structural flexibility [64]. Notably, a conserved Glu residue (Glu170 in *E. coli*) is positioned close to the signal peptide binding pocket in the plane of the membrane and potentially perturbs the bilayer structure [12, 64, 65]. Additional residues needed for TatC function reside in the cytoplasmic N-region and the first cytoplasmic loop [61, 66].

## The *B. subtilis* Tat Translocase

The Tat translocase can minimally function with just TatA and TatC [5, 67–69]. Interestingly, the Gram-positive bacterium *B. subtilis* has two minimal Tat translocases encoded by the *tatAdCd* and *tatAyCy* operons, which work in parallel and with different cargo specificities (Table 1) [5]. TatAdCd has only one known cargo protein, PhoD, which is co-expressed with the translocase under phosphate-deprived conditions [68, 70]. TatAyCy is constitutively expressed, along with its cargo proteins EfeB (YwnN), QcrA and YkuE [5, 71–74]. The third TatA gene of *B. subtilis*, *tatAc*, is constitutively expressed from another locus, and was shown to serve a non-essential function in protein translocation via the TatAyCy [5, 75].

*B. subtilis* TatAd and TatAy are bifunctional, meaning that they act at the same time as *E. coli* TatA and TatB. Interestingly, *B. subtilis* TatAd can replace TatA and TatB in *E. coli* [76], whereas TatAc expressed in *E. coli* can functionally replace TatA and TatE and form active translocases with TatCd and TatCy [77, 78]. This suggests that, despite species-specific features, the translocation mechanism employed by Tat is conserved across species [76, 79].

Structural studies on *B. subtilis* TatAd (7.4 kDa) have confirmed its ‘L-shape’ arrangement in the membrane [80–82]. By itself, TatAd oligomerizes to complexes of ~ 270 kDa and, together with TatCd (28 kDa), TatAd forms complexes of ~ 230 kDa in which TatAd is stabilized by TatCd [83–85]. Although the structure of TatAd resembles that of *E. coli* TatA, the effects of particular amino acid substitutions differ for the two proteins [47, 86]. Notably, mutagenesis of the TatAd N-terminus blocks protein translocation in *E. coli tatB* mutant cells, indicating that the N-terminal residues of TatAd are needed for TatB substitution [83].

Like TatAd, TatAy (6 kDa) has a structure similar to that of *E. coli* TatA [83, 86]. In particular, the conserved Pro2 residue in the N-terminus of TatAy and its hinge region are required for protein export [75, 86]. Complexes of TatAy alone and TatAyCy have a molecular mass of ~ 200 kDa [87]. Intriguingly, a P2A mutation leads to the formation of large fibrils composed of TatAy and TatCy, suggesting that Pro2 serves a role in the termination of complex assembly [88].

TatCd and TatCy (28/28.9 kDa) resemble *E. coli* TatC, having six transmembrane helices [87, 85]. Further, the N-terminus, the first cytoplasmic loop and the C-terminal tail of TatCd and TatCy are important for protein export, but the relevance of different conserved residues depends on the cargo [89, 90].

TatAc (6.7 kDa) of *B. subtilis* shares significant sequence similarity with *E. coli* TatE, and it can actually form active Tat complexes with TatA and TatB, or with TatCd and TatCy when expressed in *E. coli* (Table 1) [75–78, 87]. Nevertheless, TatAc cannot replace TatAd or TatAy for protein translocation in *B. subtilis*, where it was shown to assist protein translocation by TatAyCy [75].

## TatA and TatA/BC Complexes

While the Tat system can handle cargo proteins of up to 150 kDa [91], the Tat components are much smaller. This implies that they need to assemble into larger complexes that can facilitate membrane passage of larger cargo proteins [92]. Indeed, two types of Tat complexes were identified, namely TatA(B)C and TatA. In *E. coli* and thylakoids, membrane-embedded TatBC complexes are believed to bind cargo proteins, whereas recruitment of TatA complexes is required to facilitate their membrane passage [93–95]. In *B. subtilis*, the cargo receptor function of TatBC complexes is fulfilled by TatAdCd or TatAyCy complexes. Notably, the TatA complexes by themselves, especially those of *B. subtilis* (Table 1), are too small and homogeneous to allow passage of most cargo proteins [68, 77, 78]. The TatA-TatA/BC assemblies are thought to disassemble upon completed cargo translocation [96].

As shown by cross-linking studies, within TatBC complexes, TatC is first to interact with the N-region of a twin-arginine signal peptide [94, 97, 98]. Subsequently, deep insertion of the signal peptide into TatC will follow, leading to interaction of the H-domain with the transmembrane segment of TatB [54, 94, 99]. In turn, this leads to exposure of the signal peptidase cleave site in the C-region to signal peptidase on the *trans*-side of the membrane [63, 100–102]. Intriguingly, several lines of evidence, suggest that more than one cargo protein can be bound by assemblies of seven TatBC copies [60, 103–105]. Within these TatBC assemblies, TatC monomers have two TatB contact sites [61, 99, 102, 106, 107]. Further, the transmembrane segment of TatB appears to be positioned close to the site where translocase oligomerization is initiated by TatA, which suggests that TatB could serve as a regulatory surrogate of TatA [108–110]. The latter would be in line with the fact that TatB is absent from minimal TatAC translocases as encountered in *B. subtilis*. Furthermore, cross-linking analyses show that cargo docking via the signal peptide leads to conformational changes that rearrange TatC’s binding site for TatA and TatB [52]. Binding of a signal peptide changes the arrangement of TatC from head-to-tail to tail-to-tail [106].

TatBC complexes contain small amounts of TatA that may serve as points of TatA nucleation for forming the active translocase [111, 112]. Most TatA molecules are, however, present in TatA complexes. The TatA complexes of *E. coli* are very heterogeneous, ranging from 100 to 500 kDa with intermediate size intervals of ~ 34 kDa [48, 54, 113–115]. In contrast, TatAc, TatAd and TatAy complexes in *Bacillus* are much smaller with molecular masses of ~ 100, ~ 270 and ~ 200 kDa, respectively [76, 77].

## Tat Translocation Mechanism

Despite almost three decades of research, the Tat translocation mechanism is still incompletely understood. As outlined above, cargo translocation is initiated at TatA/BC complexes and then facilitated by TatA [113]. This may involve either pore formation [116] or membrane weakening [43, 117].

Based on low-resolution EM images, it was proposed that TatA complexes have a pore of 8.5–13 nm that might accommodate cargo proteins of varying size [116, 118]. This pore would be closed by a lid at the cytoplasmic side membrane, resembling a ‘trap door’, which could swing open with the help of a conserved Gly residue in the hinge region of TatA to allow cargo passage [118, 119]. In this scenario, after cargo docking onto TatBC, TatA would be recruited to form an oligomeric ring conforming to the size of the cargo protein [120, 121]. Although this model appears attractive, the trap door concept has not been confirmed in other studies [46, 48, 122]. Moreover, complexes of the TatA paralogue TatE (50–110 kDa) appear too small for pore formation [43].

More recently, it was proposed that TatA complexes might serve to weaken the membrane [48, 106, 117, 123]. This would relate to the relatively short transmembrane domain of TatA that can locally restrict the membrane thickness. This membrane weakening would only occur upon cargo binding and interaction of the mature part of the cargo protein with the amphipathic helix of TatA [94, 99, 123–126]. In the absence of cargo, the membrane weakening would not take place as immersion of the amphipathic helix of TatA in the membrane would preserve the membrane integrity, as was shown for the thylakoidal TatA [122].

As mentioned above, protein translocation via Tat is exclusively driven by the proton-motive force, which consists of the ΔpH and the electric potential Δψ across the membrane [127]. Early studies into the energetics of Tat were performed in vitro with the plant thylakoid system. In the presence of light and a ΔpH, but in the absence of nucleotides, the photosystem II oxygen-evolving Tat cargo protein tOE23 was still exported [128]. In addition, a phage shock protein PspA, involved in maintaining the proton-motive force, was found to increase Tat translocation in bacteria [129]. However, in vivo studies in the green alga *Chlamydomonas reinhardtii* showed that the system can still transport proteins without a thylakoidal ΔpH, which can be explained by the fact that the Tat pathway can use both the ΔpH and Δψ [130, 131]. As a consequence of this equivalency, an antiporter mechanism was suggested where a coupling of H^+^ flow and protein transport has been suggested [132]. Of note, the counterflow of protons necessary for Tat protein export was estimated to amount about 7.9 × 10^4^ protons per molecule [133]. This is equivalent to 10,000 ATP molecules, 3% of the energy produced by a chloroplast, so it is a considerable cost to the cell.

With regards to individual steps of the translocation mechanism, in vitro studies have shown that the proton-motive force is not required for protein targeting or protein binding to TatBC, but for the more advanced binding stages and oligomerisation of TatA [94, 134]. For thylakoids it was proposed that the ΔpH could potentially protonate TatA (Tha4) at the Glu10 residue, making it energetically feasible to move up in the membrane to its docking site in TatC (Gln234) [112]. However, in an earlier study this Glu10 residue was replaced with Gln, as well as with Ala or Asp, and all of these changes severely reduced the ability of TatA to facilitate protein transport [135]. While this shows the importance of the Glu10 residue and a negative charge at this position for translocation activity, it is not certain whether this implies a role of Glu10 in sensing the thylakoidal luminal pH through protonation, or whether Glu10 forms a salt bridge with a basic residue somewhere else [135]. It is also still unclear how the assembly of TatA in *E. coli* is facilitated by the proton-motive force, as it has been shown through in vitro studies that the transport driving force is largely provided by the Δψ [136]. In fact, these studies indicate that two distinct Δψ-dependent steps drive protein transport: a first step would involve a ∆ψ of relatively high magnitude that may be short-lived, and a second step of longer duration would require a ∆ψ of relatively low magnitude. When the ∆ψ was increased, so was the transport speed [94, 136]. This raises the question, how exactly the ∆ψ drives protein transport via Tat in *E. coli* and why this is apparently different in thylakoids, where the ΔpH represents the driving force for protein transport. A conceivable scenario is that movement of certain charged regions within the membrane-embedded *E. coli* Tat proteins could be induced by a ∆ψ, whereas this process would be induced by the ΔpH in the chloroplast thylakoidal membrane. Altogether, it is presently still unclear whether a potential across the membrane drives charge movements or whether proton transport by Tat takes place.

## Chaperoning of Tat Cargo Proteins

One of the major hallmarks of the Tat pathway is its ability to selectively transport fully folded cofactor-containing proteins. To this end, the system involves different mechanisms. Translocation of particular cargo proteins requires the aid of dedicated chaperones, known as redox enzyme maturation proteins (REMPs) [137, 138]. An example of a Tat cargo protein involving a REMP for export is the oxidoreductase trimethylamine-*N*-oxide (TMAO) reductase (TorA; Fig. 1). This enzyme is encoded by the *torCAD* operon, where *torA* encodes the TMAO reductase, *torC* its haem-binding quinol oxidase and *torD* its REMP. In particular, TorD recognizes and binds the h- and c-regions of the TorA signal peptide most likely as a dimer [139–141]. Following signal peptide binding, TorD guides TorA export via Tat in a GTP-dependent manner. In this scenario, the affinity of TorD for GTP increases upon signal peptide binding, and GTP presumably controls cycles of signal peptide binding and release of TorD, thereby preventing premature protein degradation, coordinating cofactor assembly and foreseeing other maturation steps, such as membrane targeting and interaction [139, 140]. This coordination occurs until the pre-protein interacts with the Tat machinery.

## Proofreading of the Folding State of Tat Cargo

The proofreading exhibited by the *E. coli* and *B. subtilis* Tat pathways is highly stringent to ensure misfolded proteins are not exported. Thus, the Tat complex rejects and may sometimes even degrade cargo proteins within the cytosol, although such degradation may also occur independently of the Tat system [142–144]. To note, the thylakoidal Tat system seems to have a less stringent ‘proofreading’ system as unfolded proteins are also imported [37].

To explore mechanisms of Tat proofreading, particular attention has been attributed to cofactor insertion. The native *E. coli* Tat cargo proteins NrfC and NapG were mutated to prevent their central cofactor FeS binding. Indeed this alteration blocked export [143]. The *B. subtilis* Rieske iron-sulphur cluster protein QcrA was also mutated to either stop cofactor binding or disulphide bond formation [145]. Here, a proofreading hierarchy was uncovered: mutant’s defective in disulphide bonding were quickly degraded, whereas those defective in cofactor binding accumulated in the cytoplasm and membrane. Two heme-binding proteins have also been investigated for proofreading. First, cytochrome C was shown to require heme insertion for export [146]. Subsequently, proofreading was investigated with the synthetic BT6 maquette protein, which binds two hemes and is Tat-dependently secreted in *E. coli* when provided with a TorA signal peptide [147]. His residues in BT6 were replaced with Ala to prevent heme binding. This showed that export was completely blocked if heme binding was completely prevented. Binding of one heme by BT6 allowed some export, whereas good export was observed when two hemes were bound [147]. Altogether, these findings suggest that Tat can somehow sense a protein’s conformational stability.

The requirement for conformational stability was further studied in vivo and in vitro with non-native Tat cargo proteins, such as *E. coli* PhoA and scFv or Fab antibody fragments. Export of these proteins only occurred in oxidizing conditions allowing disulphide bond formation prior to their interaction with the Tat machinery [40]. Nevertheless, some proteins provided with a twin-arginine signal peptide, like human growth hormone (hGH), scFv’s and interferon α2b, were exported to the periplasm without their disulphide bonds formed [148]. For hGH it was shown that this protein can form a near native state in absence of its two disulphide bonds. This is reminiscent of observations on the CueO protein of *E. coli*, which can still be exported via Tat without its bound copper cofactor. This probably relates to the fact that CueO without bound copper is structurally close to identical to CueO with bound copper [149].

Several studies in both bacteria and plants have used varying lengths of FG repeats from the yeast nuclear pore protein Nsp1p to probe the structural constraints for Tat-dependent export. These repeats intrinsically lack structure and are hydrophilic. Fused to a Tat signal peptide, export studies demonstrated that with increasing protein length, the translocation efficiency decreased: segments of 100–120 amino acids were tolerated, but a short hydrophobic stretch stopped export [150, 151]. Unstructured linkers were also placed between the signal peptide and the N-terminus of a mature Tat cargo protein and, surprisingly, an unstructured linker length of 110 amino acids was exported [152]. These findings imply that, despite the generally strict folding requirement for Tat cargo proteins, short unstructured polypeptide regions can be tolerated in particular protein contexts.

A recent study used scFv mutants [153], which were structurally defined, to identify what the *E. coli* Tat machinery recognizes as ‘unfolded’ and rejects for export [154]. Tat tolerated significant changes in hydrophobicity and charge, but did not export the scFv with an unstructured tail or without cytoplasmic disulphide bond formation via the so-called CyDisCo system. CyDisCo comprises the yeast mitochondrial thiol oxidase Erv1p *plus* the human protein disulfide isomerase PDI that, together, confer the ability to catalyse cytoplasmic disulphide bond formation.

Since it is still unclear what exactly the Tat complex rejects as misfolded, a key question is how the Tat complex rejects certain proteins. Tat proteins, misfolded or not, both interact with the Tat translocase. For example the PhoA protein provided with a twin-arginine signal peptide has been co-purified with TatBC [41]. This gave rise to the idea Tat does not innately have an inbuilt ‘proofreading’ mechanism, but rather an efficient degradation system that clears the Tat translocase. Indeed, the *B. subtilis* protease WprA was shown to interact directly with the Tat machinery and to be essential for protein secretion via TatAyCy [155, 156].

Lastly, in vitro site-specific photo cross-linking experiments revealed that unfolded TorA-PhoA associated with the Tat translocase, and that the interaction with the TatBC receptor site was perturbed as if the cargo was not correctly inserted into the binding socket [157]. This invoked the TatBC complex in proofreading of the cargo protein. This view is consistent with the identification of so-called quality control suppression (QCS) mutations within *E. coli* TatABC, which gave rise to less stringent proofreading [158]. The majority of these QCS mutations were confined to the unstructured or loop regions of TatABC, showing that proofreading at some level is undertaken by the Tat translocon.

## Conclusion

In recent years, the core components of the Tat protein translocation systems have been identified, biochemically characterized and structurally defined. Yet, the precise mechanism by which Tat translocates proteins across the bacterial cytoplasmic membrane is still elusive due to the fact that high-resolution structural data of protein-translocating Tat complexes is currently missing. It can be anticipated that with the advent of novel high-resolution techniques for structural analyses of large protein complexes many of the so far unanswered fundamental questions in the Tat field can be tackled and answered.
